# Perceived facilitators and barriers by esophageal cancer survivors participating in a post-treatment exercise program

**DOI:** 10.1007/s00520-023-07769-5

**Published:** 2023-05-06

**Authors:** Jonna K. van Vulpen, Lenja Witlox, Alida C. Methorst-de Haan, Anouk E. Hiensch, Richard van Hillegersberg, Jelle P. Ruurda, Grard A.P. Nieuwenhuijzen, Ewout A. Kouwenhoven, Peter D. Siersema, Anne M. May

**Affiliations:** 1 Department of Radiation Oncology, University Medical Center Utrecht, Utrecht University, Heidelberglaan 100, 3584 CG Utrecht, The Netherlands; 2 Julius Center for Health Sciences and Primary Care, University Medical Center Utrecht, Utrecht University, P.O. Box 85500, STR 6.131, 3508 GA Utrecht, The Netherlands; 3 HU University of Applied Sciences, Heidelberglaan 15, 3584 CS Utrecht, The Netherlands; 4 Department of Surgery, University Medical Center Utrecht, Utrecht University, Heidelberglaan 100, 3584 CG Utrecht, The Netherlands; 5 Department of Surgery, Catharina Hospital, Michelangelolaan 2, 5623 EJ, Eindhoven, The Netherlands; 6 Department of Surgery, ZGT Hospital, Zilvermeeuw 1, 7609 PP Almelo, The Netherlands; 7 Department of Gastroenterology and Hepatology, Radboud University Medical Center, PO Box 9101, 6500 HB Nijmegen, The Netherlands

**Keywords:** Cancer survivorship, Esophageal cancer, Exercise, Quality of life, Adherence, Facilitators and barriers

## Abstract

**Purpose:**

Participation in a post-treatment exercise program improves cardiorespiratory fitness and aspects of quality of life for esophageal cancer survivors. For optimal effects, high adherence to the exercise intervention is important. We assessed which facilitators and barriers to exercise adherence are perceived by esophageal cancer survivors, who participate in a post-treatment exercise program.

**Methods:**

The current qualitative study was performed within the randomized controlled PERFECT trial, in which we investigated effects of a 12-week supervised exercise program with moderate-to-high intensity and daily physical activity advice. Semi-structured interviews were conducted with patients randomized to the exercise group. A thematic content approach was used to derive perceived facilitators and barriers.

**Results:**

Thematic saturation was reached after inclusion of sixteen patients. Median session attendance was 97.9% (IQR 91.7–100%), and relative dose intensity (compliance) to all exercises was ≥90.0%. Adherence to the activity advice was 50.0% (16.7–60.4%). Facilitators and barriers were captured in seven themes. The most important facilitators were patients’ own intention to engage in exercise and supervision by a physiotherapist. Barriers were mainly experienced in completion of the activity advice, and included logistic factors and physical complaints.

**Conclusions:**

Esophageal cancer survivors are well capable to attend a moderate-to-high intensity post-treatment exercise program, and to fulfill the exercises according to protocol. This is facilitated by patients’ own intention to engage in exercise and supervision of the physiotherapist, and only minimally affected by barriers as logistic factors and physical complaints.

**Implications for cancer survivors:**

When implementing postoperative exercise programs in clinical care, it can be useful to be aware of perceived facilitators and barriers of cancer survivors in order to achieve optimal exercise adherence and maximize beneficial exercise effects.

**Trial registration:**

Dutch Trial Register NTR 5045

## Introduction

Patients who have been treated for cancer may experience cancer-related and treatment-related side effects impairing quality of life in the short and long term. Participation in (post-treatment) exercise programs has been proven to beneficially affect several health-related outcomes, such as fatigue, physical fitness, and quality of life [1–5]. Also in esophageal cancer survivors, we showed that a 12-week combined aerobic and resistance exercise program improves several aspects of quality of life, physical fitness, and fatigue [6].

Like any medical intervention, uptake of the exercise program (adherence) by the patient is important to achieve optimal effects. For exercise programs, it is therefore important that participating patients are present at the prescribed exercise sessions (attendance). Also, benefits of an exercise program may be largest if patients fulfill all components of the exercise program according to the exercise protocol (compliance or relative dose intensity (RDI)). Attendance and RDI are the two components that are united in the broader concept “adherence” [7, 8]. Unfortunately, maintaining high adherence rates can often be challenging [9].

In the Physical ExeRcise Following Esophageal Cancer Treatment (PERFECT) trial, we randomized esophageal cancer survivors between usual care and a 12-week post-treatment exercise program. The exercise program consisted of both aerobic and resistance exercises, performed at moderate-to-high intensity and individualized to the patients’ fitness level before start of the program [10]. Although treatment of patients with esophageal cancer is extensive (typically neo-adjuvant chemoradiation followed by esophagectomy) and patients frequently have comorbidities and impaired fitness levels (~40% comorbidities and mean peak VO_2_ of 22.5 ml/min/kg (SD 5.4) in our study), adherence to the PERFECT exercise program was high [6].

When applying exercise programs in regular clinical cancer care, adherence rates ideally should be comparably high as found in this trial. An understanding of which elements help or hamper patients to complete the exercise program as prescribed can support in designing exercise programs for clinical practice. Information on barriers for attending exercise sessions or not fulfilling specific components of the exercise program can help to improve the program and decrease dropout rates, while information on facilitators can indicate which aspects of the program should be retained or even extended for optimal adherence. For the current study, we performed semi-structured telephone interviews with patients participating in the PERFECT exercise program, to identify facilitators and barriers to exercise adherence.

## Methods

The current research question was investigated within the multicenter randomized controlled PERFECT study (NTR5045). The PERFECT study was approved by the Medical Ethics Committee of the UMC Utrecht and the local Ethical Boards of participating hospitals. Informed consent was obtained from all participants included in the study. A detailed description of the trial design has been published elsewhere [10]. In brief, the primary aim of the trial was to investigate the effects of a 12-week combined aerobic and resistance postoperative exercise program on quality of life in esophageal cancer survivors. Inclusion criteria were as follows: 4 weeks to 1 year after hospital discharge following radical esophagectomy; no distant metastases; age ≥18 years; able to read and understand the Dutch language; physically inactive (≤ 150 min per week of moderate-to-high intensity exercise at time of inclusion); not involved in another supervised exercise program; Karnofsky Performance Status ≥60; able to walk ≥60 m; and no contra-indications for physical activity (as assessed through the Physical Activity Readiness Questionnaire (PAR-Q) [11]). After completion of baseline measurements, participants were randomized to either the exercise or usual care arm in a 1:1 ratio. For those randomized to exercise, a physiotherapist was identified and trained to supervise the 12-week exercise program according to the intervention protocol. For the current research aim, we were interested in perceived barriers and facilitators by participants who were allocated to the exercise arm of the PERFECT study. The first sixteen consecutive patients who were randomized to the exercise arm were approached and all sixteen patients agreed to participate in the interview.

### Intervention

The 12-week exercise program consisted of two 1-h exercise sessions per week. To keep travel distances to a minimum, the exercise program was supervised in a general physiotherapist practice or hospital close to the patient’s home address. The training was individualized to the patient’s fitness level at baseline, as determined with cardiopulmonary exercise testing for aerobic training and repetition maximum (RM) testing for resistance training. The exercise protocol was standardized and aimed at training on a moderate-to-high intensity (Table 1).

**Table 1:** Exercise protocol

Week	Aerobic training	Resistance training
1-3	15-20 min 40-60% HRR	One set of 20-25 repetitions at 20-RM weight for each exercise*
4-8	15-20 min 60-70% HRR + 5-10 min 70-89% HRR	
9-12	10 min 60-75% HRR + Interval training: 10 × 30 s vigorous to maximal exercise, alternated with a 1-min active rest	Two sets of 15-20 repetitions at 15-RM weight for each exercise**

Protocol of the exercise intervention prescribed in the PERFECT study

*HRR* heart rate reserve; *RM* repetition maximum

*Rowing, bench press, squat, shoulder press, biceps curl, lunges, calf-raises, triceps extension, abdominal crunch

**Rowing, bench press, squat, shoulder press, biceps curl, triceps extension, abdominal crunch/hoover

In addition to the supervised exercise program, patients were encouraged to be physically active for at least 30 min per day, on all remaining days of the week. During an intake session, the physiotherapist supported the patient to set appropriate exercise goals of moderate intensity, in agreement with the patient’s fitness and preferences. All daily activities were documented in an exercise log, which was discussed with the physiotherapist every 2 weeks to evaluate achievement of exercise goals and potentially set new ones.

### Outcome measures

#### Adherence

To monitor session attendance, each physiotherapist reported on presence of the patient at each training session in a case report form. In addition, to monitor the relative dose intensity for the different exercise components, heart rates and duration were documented for the aerobic training and resistance and number of sets and repetitions of each muscle strength exercise for the resistance training. Adherence to the exercise advice of being physically active for at least 30 min per day and adherence to the Dutch Physical Activity Guidelines, i.e., engaging in at least 150 min of exercise per week^12^, were determined through evaluation of the exercise logs.

#### Facilitators and barriers

A topic list and an interview protocol were prepared before the start of the interview series, after examination of relevant literature and discussion within the study team. All semi-structured interviews, except one, were performed within 14 days of completing the exercise program and were audio-recorded and transcribed verbatim (LW and ACM).

### Data analysis

Descriptive statistics were used to summarize characteristics of the study population. Attendance rates were computed as the number of supervised exercise sessions attended, divided by the number of sessions prescribed. The RDI (i.e., compliance) was calculated as the ratio of total completed to total planned cumulative dose for the exercise program. The percentage of weeks in which patients adhered to the exercise advice of being physically active for at least 30 min/day was calculated, as well as the percentage of weeks in which patients adhered to the Dutch Physical Activity Guidelines (engaging in at least 150-min exercise per week). Quantitative data were analyzed using IBM SPSS Statistics^TM^ (version 25.0, IBM, Armonk, New York).

Transcribed interviews were imported in NVivo 11 for thematic analysis. All transcripts were coded by two researchers independently (LW and JKV), who met regularly to resolve differences in coding for each transcript. Coded text was compared across transcripts to identify broader themes, capturing patients’ perceptions on barriers and facilitators. Thematic saturation was reached after 16 interviews, i.e., the final three interviews did not generate new themes.

## Results

The PERFECT study included patients from March 2015 to January 2019 and included a total of 120 patients (of which 61 patients were randomized to the exercise program and 59 patients to usual care). Between March 2015 and December 2015, 16 patients were allocated to the exercise program, who were also included in the current study. Baseline characteristics of these patients are summarized in Table 2. The majority was male (81.3 %), mean age was 62.6 ± 7.2 years, and median time after surgery was 98 days (interquartile range (IQR) 72–169). Baseline characteristics of these 16 patients were comparable to those of all PERFECT participants [6].

**Table 2:** Baseline characteristics

	Patients (*N* = 16)
Age, mean (SD) years	62.6 (7.2)
Time after surgery, median (IQR) days	98 (72–169)
Sex, *n* (%)	
Male	13 (81.3)
Female	3 (18.8)
Education, *n* (%)	
Low	5 (31.3)
Medium	8 (50.0)
High	3 (18.8)
Marital status, *n* (%)	
Couple	14 (87.5)
Single	2 (12.5)
Tumor type, *n* (%)	
Adenocarcinoma	13 (81.3)
Squamous cell carcinoma	3 (18.8)
Comorbidities, *n* (%)	
Yes	6 (37.5)
No	10 (62.5)
Type of surgery, *n* (%)	
Open esophagectomy	2 (12.5)
Minimally invasive esophagectomy	14 (87.5)
BMI, mean (SD) kg∙m^−2^	24.9 (4.0)
Peak VO_2_, mean (SD) ml/min/kg	21.9 (6.8)

*SD* standard deviation; *IQR* interquartile range; *BMI* body mass index; *VO*_*2*_ oxygen uptake

### Adherence

Attendance in this group of 16 patients was 97.9% (IQR 91.7–100%) (Table 3). All components of the aerobic exercise part and resistance exercise part reached a median RDI of >90%. With regard to the advice to be physically active for ≥30 min per day, all days of the week, RDI was 50.0% (IQR 16.7–60.4%). RDI for being active 5 days of the week (Dutch Physical Activity Guidelines [12]) was 91.7% (IQR 75.0–100%) (Table 3).

**Table 3:** Adherence

	Adherence, median % (IQR)
Attendance	97.9 (91.7–100)
Relative dose intensity	
Aerobic exercise*	
*Moderate intensity*	
*Duration*	95.8 (90.0–100)
*Intensity*	93.8 (90.0–99.8)
*High intensity*	
*Duration*	95.0 (80.0–100)
*Intensity*	94.7 (79.1–100)
*Interval training*	93.8 (87.5–100)
Resistance exercise	
*Legs*	92.0 (88.1–99.6)
*Arms*	95.2 (87.6–98.0)
*Shoulders*	95.6 (89.3–97.3)
*Back*	94.8 (90.1–99.4)
*Core*	90.7 (80.8–93.8)
Physical activity advice	
* Percentage of weeks being active on 7 days/week*	50.0 (16.7–60.4)
* Percentage of weeks being active on 5 days/week*	91.7 (75.0–100)

Attendance is defined as presence at the exercise sessions, and relative dose intensity is defined as fulfilling all components of the exercise program according to protocol

*IQR* interquartile range

*Moderate intensity training was performed at 40-75% of the heart rate reserve; high intensity training was performed at 70-89% of the heart rate reserve; interval training consisted of 10 × 30 s vigorous to maximal exercise, alternated with a 1-min active rest (see Table 1)

### Facilitators and barriers

We identified 7 overarching themes that captured patients’ perceived facilitators and barriers during their participation in the exercise program: intention to engage in exercise, supervision, psychosocial context, physical condition, logistics, content of the exercise program, and information about the exercise program and training progress (Table 4). The theme “psychosocial context” captured facilitators only, whereas the remaining themes were considered to include both facilitators and barriers.

**Table 4:** Overarching themes that capture patients’ perceived facilitators and barriers to exercise adherence

Themes	Facilitators	Barriers
Intention to engage in exercise	Motivation and commitment Personal goals Fun Past positive exercise experiences	Emotions Limited body confidence Not feeling like it
Supervision	Motivating influence Expertise Mental support Obligation	Absence physiotherapist
Psychosocial context	Social support Embedding in daily life	-
Physical condition	Experiencing progress	Esophageal dilation therapy General physical complaints Pre-existing injuries
Logistics	Availability of equipment Location Weather circumstances Time Flexibility appointments	Conflicting activities National holidays and vacation Weather circumstances Inflexibility appointments
Contents exercise program	Individualization of exercises Diversity of exercise components	Limited variation in exercises Intensified training load
Information about the exercise program and training progress	Knowledge on training schedule Progress Quantification of physical activity	Ignorance of exercise side effects

#### Facilitators

First, patients reported their own intention to engage in exercise as the most important facilitator to adhere to the exercise program. Most patients were highly motivated to contribute to their own recovery and were convinced that the exercise program would help them to improve their health. Their own personal goals of improving condition, strength, and health increased their commitment. Also, previous positive training experiences, e.g., pre-operatively, had a positive influence on fulfillment of the exercise program. In addition, some indicated exercising to be a pleasant activity, which made it easier to adhere.

Second, supervision by the physiotherapist was considered to be a strong facilitator. Within this theme, several aspects had beneficial influence on exercise adherence. With the physiotherapist encouraging the patients while performing the exercises, patients felt motivated to challenge themselves, even when they had a hard time with the exercises. In addition, some reported to experience mental support by the physiotherapists and many especially valued the medical expertise and knowledge on correct performance of all exercises, which made them feel comfortable to perform the prescribed program. Last, patients felt obligated to go to the two weekly appointments with the physiotherapist, which was for many of them a great help to bring up the necessary discipline to exercise (quote 1).

1. “Yes, because it is in any case very important to exercise and if that is compulsory, because you have to do it, you also put aside hesitations to actually doing it. I mean, if something is obligatory, you commit to it, and that in itself is a very good thing.”

With regard to the content of the exercise program, patients indicated that RDI was promoted if the components were individualized to patients’ preferences and if exercises were adjusted in case of pre-existing injuries. Also, variation in exercises, such as alternating leg and arm strength exercises, made the exercise program more attractive to comply to. Patients further reported to feel encouraged when they experienced progress in their physical condition and daily activities (quote 2).

(2) “I loved it. It made it most clear to me that I was on the mend, it was measurable, I immediately noticed that my energy was improving. Even when I was really tired at some day, I knew: When I have been to physiotherapy, I will feel better.”

In line with experiencing progress themselves, patients indicated that adherence was promoted by receiving information on their training progress, i.e., quantification of training results, as well as information on the training schedule. Regarding the physical activity advice, collecting information through apps with pedometers (on their own initiative) helped three patients to achieve the prescribed guideline of activity per day. In addition, the psychosocial context influenced adherence to the home-based exercise guideline. Performing activities together with a partner and embedding the activities in daily life, e.g., walking with the dog, supported performing physical activity on a regular base.

Last, logistic factors such as short distance to the training facility, having enough time for exercising (e.g., being retired), and flexibility of the appointments (e.g., in case of conflicting activities) were all mentioned as facilitators. Furthermore, the availability of equipment and good weather circumstances made it easier to fulfill the physical activity advice.

#### Barriers

The most important barriers were captured by the themes “physical condition” and “logistics”. Esophageal dilation, commonly performed in post-esophagectomy patients, hampered both attendance and RDI: Due to the hospital visits, appointments with the physiotherapist could be affected and patients needed some time to fully recover from the treatment and sedation (quote 3).

(3) “Well, every time I had to go back to the hospital and they had to dilate me [esophagus]. Yes, that day itself you are always... Because 24 hours prior you are not allowed to eat anything. The day itself you are travelling to the hospital all day. Sometimes the day after, you felt a bit drowsy due to the sedation. So, sometimes it was more difficult to keep going with the program.”

In few other cases, patients could not comply with the exercises because of other physical complaints, such as dizziness, not feeling well, or pre-existing injuries. Regarding logistics, for the supervised part, mainly national holidays and vacation, and impossibility of changing the training schedule to other days or daytimes, were mentioned as barriers. For the exercise advice, weather circumstances (rain for some, high temperatures for others) influenced adherence, as well as conflicting activities, such as doing the housekeeping and job obligations. To a lesser extent, absence of the physiotherapist could act as a barrier.

When patients felt psychologically unhappy or when they just did not feel like exercising, it was more difficult to adhere to the program. Also, limited body confidence, which had developed after surgery, resulted in an insecure feeling when performing the exercise components. Feelings of insecurity were also caused by a lack of knowledge on exercise side effects, e.g., muscle pain. Last, regarding the contents of the program, a perceived lack of variation in exercises was a barrier to some, and intensification of the training load to others.

## Discussion

In this qualitative study, we observed that esophageal cancer survivors who participate in a moderate-to-high intensity exercise program in the first postoperative year are well capable to attend the exercise sessions and comply to the prescribed aerobic and resistance exercises. Perceived facilitators and barriers to adhere to the exercise program were captured in seven main themes: intention to engage in exercise, supervision, psychosocial context, physical condition, logistics, content of the exercise program, and information about the exercise program and training progress.

According to the exercising esophageal cancer survivors, especially their own intention to engage in exercise was helpful to fulfill the exercise program as prescribed. While this finding represents the patient’s perspective, it is also supported by previous systematic reviews that quantitatively assessed determinants of exercise adherence in cancer survivors (not including esophageal cancer survivors). A review that specifically focused on motivational and behavioral variables identified exercise stage of change (i.e., the stages people move through when modifying behavior, derived from the transtheoretical model of behavior change), intention, and perceived behavioral control as moderately strong predictors for adherence to exercise intervention programs [13]. In another review, a best evidence synthesis was performed, taking into account demographic, clinical, psychological, physical, social, and environmental factors as determinants of exercise adherence. Here, the authors found moderate evidence for a positive association between exercise history and exercise adherence [14]. In line with this review, participants in our study indicated past positive exercise experiences to be a facilitator. Such positive experiences can either be gained prediagnosis, or as part of the nowadays frequently applied prehabilitation/enhanced recovery after surgery (ERAS) programs [15–17].

For patients lacking a positive intention towards engaging in exercise, it might be beneficial to motivate exercise behavior, e.g., by incorporating principles of behavioral change techniques [18]. Moreover, patients tend to be physically more active if they receive exercise recommendations from their medical specialist. Therefore, involvement of medical specialists can have a critical role in physical activity promotion and motivating patients to overcome perceived barriers [19, 20]. While surveys show that health care providers in oncology care teams in general acknowledge benefits of physical activity for cancer survivors [21] and consider it to be part of clinical cancer care [22], only half of them promote physical activity on a regular base [20]. The main barriers among health care providers are a lack of knowledge regarding the beneficial effects of exercise after a cancer diagnosis, the lack of awareness regarding the availability of exercise programs, a lack of time, and uncertainty about safety of physical activity for cancer survivors [21, 23]. Possible strategies to overcome these barriers include education of health care providers (e.g., in evidence regarding exercise benefits and safety, and motivational interviewing), comprehensive guidelines [24–26] to help to guide medical specialists’ recommendations regarding exercise, and availability of straightforward referral tools [21–23, 27].

Supervision of a physiotherapist was another frequently mentioned facilitator for achieving a high adherence with the exercise program. In our study, we worked together with trained physiotherapists, preferably with expertise in oncology. Patients reported that physiotherapists helped them to commit to the program not only by providing medical expertise, but also by motivating them, delivering mental support, and creating the feeling of an (positive) obligation to exercise. Benefits from supervised exercise programs have previously been shown in other disciplines, such as intermittent claudication [28]. The multifaceted role of physiotherapists also came out in a previous qualitative exploration among men with prostate cancer, who reported that their supervising physiotherapist had played a role in the success of the exercise program by serving as an educational resource and support provider [29]. Furthermore, a trial comparing the effects of two different exercise programs during chemotherapy for breast cancer showed that more patients were adherent to a supervised moderate to high intensity exercise program (~60%) than to a low intensity home-based, self-managed program (~50%). Moreover, one of the most frequently patient reported suggestions for improving the home-based program was to add more encouragement and support as part of the program. Also, for the supervised exercise program, patients suggested to include more one-on-one supervision [30].

Patients only minimally reported barriers while participating in the exercise program, which explains the high adherence rates for both the aerobic and resistance parts of the program. Adherence to the daily physical activity advice appeared to be more difficult. This has also been observed in other patient groups, when combining a supervised part with a home-based physical activity advice [31–33]. Logistic barriers such as weather circumstances and conflicting activities played a role. The psychosocial context (i.e., social support and embedding of physical activity in daily life) on the other hand was an important motivational influence for the home-based part and might help patients being less limited by their (logistic) barriers. Suggestions to achieve this can be to find an exercise buddy and use a smartwatch or phone with a pedometer or activity tracker. The development of telerehabilitation, i.e., receiving guidance for physical activity in the home-based setting via eHealth, may also be interesting in this respect [34–36]. Physical complaints were identified to be a barrier both to session attendance and RDI. Although these complaints are not easily modifiable, patients reported that flexibility, both in terms of appointments and of program contents, could be helpful in this respect.

A limitation of the current study is that we used exercise logs, kept by the patients, to record adherence to the physical activity advice. This could on the one hand have resulted in underreporting of physical activities, due to patients forgetting to log their activities. On the other hand, it could have resulted in overreporting of physical activity as a result of patients showing socially desirable behavior in filling out the exercise logs. Furthermore, our results are only generalizable to patients already participating in exercise, and do not provide information on facilitators or barriers for starting exercise in the whole group of esophageal cancer survivors. While we could not extract differences in experienced barriers and facilitators from the interviews, it should be recognized that the amount and type of barriers and facilitators may differ depending on the moment of initiating an exercise program. Considering saturation as the leading concept for sample sizes when using qualitative interviews [37], the sample size was sufficient for the extraction of themes from the interviews. A strength of our study is the extensive reporting of both attendance and RDI, which required a consistent logging of all exercises by the supervising physiotherapists as well as a strict monitoring by the study team. The qualitative data obtained through semi-structured interviews provided a comprehensive insight into patient perceived facilitators and barriers.

In conclusion, esophageal cancer patients after curative treatment are well capable to attend a moderate-to-high intensity exercise program and to fulfill the resistance and aerobic exercises according to protocol. They report their adherence to be facilitated by their own intention to engage in exercise and supervision of the physiotherapist. Completion of the exercise program was only minimally affected by perceived barriers as logistic factors and physical complaints. Information on the facilitators and barriers can help in optimizing adherence to exercise in clinical practice, to achieve maximum results for post-treatment esophageal cancer patients.
